# Contrast-enhanced ultrasonography for differential diagnosis of adnexal masses

**DOI:** 10.3389/fonc.2022.968759

**Published:** 2022-10-20

**Authors:** Weihui Shentu, Yin Zhang, Jiaojiao Gu, Fa Wang, Wei Zhao, Chunmei Liu, Zimei Lin, Yao Wang, Chen Liu, Yunyu Chen, Qiyun Fan, Hongying Wang

**Affiliations:** ^1^ Department of Medical Ultrasonics, Guangzhou Women and Children’s Medical Center, Guangzhou Medical University, Guangzhou, China; ^2^ Department of Medical Ultrasonics, The Second Affiliated Hospital of Zhejiang University School of Medicine, Hangzhou, China

**Keywords:** contrast-enhanced ultrasound, adnexal mass, ovarian cancer, benign, malignant

## Abstract

**Background:**

Quantitative contrast-enhanced ultrasonography parameters are affected by various factors. We evaluated corrected quantitative contrast enhanced ultrasonography in differentiating benign adnexal tumors from malignant tumors.

**Methods:**

Patients with adnexal masses who underwent conventional and contrast-enhanced ultrasonography were included. Contrast-enhanced ultrasonography parameters such as base intensity, arrival time, peak intensity, time to peak intensity, ascending slope, and descending slope were measured. Corrected (time to peak intensity − arrival time) _mass/_(time to peak intensity − arrival time) _uterus_ and (peak intensity − base intensity) _mass/_(peak intensity − base intensity) _uterus_ were calculated. Lesions were confirmed by pathologic examination of surgical specimens.

**Results:**

This study included 31 patients with 35 adnexal lesions including 20 (57.10%) benign and 15 (42.90%) malignant lesions. The corrected contrast-enhanced ultrasonography quantitative parameters in lesions were statistically different between malignant and benign groups (*P*<0.05). The optimal cut-off value for (time to peak intensity − arrival time) _mass_/(time to peak intensity − arrival time) _uterus_, ascending slope, and (peak intensity − base intensity) _mass_/(peak intensity − base intensity) _uterus_, and descending slope for differentiating malignant adnexal masses from benign tumors were 1.05 (area under curve: 0.93,* P*<0.05), 1.11 (area under curve: 0.83, *P*<0.05), 0.82 (area under curve: 0.73, *P*<0.05), and −0.27 (area under curve: 0.66, *P*=0.16), with sensitivity and specificity of 93.33% and 85.00%, 86.67% and 75.00%, 86.67% and 60.00%, and 54.55% and 66.67%, respectively.

**Conclusions:**

Corrected contrast-enhanced ultrasonography parameters provide practical differential diagnosis value of adnexal lesions with high reliability for sonologists.

## Introduction

Adnexal masses are commonly encountered in daily radiology practice and occur in women of all ages. Adnexal malignancy accounts for 2.5% of all malignancies among females and 5% of all cancer-related deaths (1). Adnexal malignancy has been termed a “silent killer” because most patients present with few symptoms or are diagnosed at advanced stages (III and IV) (1, 2). While patients with late-stage disease have a high fatality rate, women with early-stage disease have an overall 5-year survival of approximately 93% (1, 3). Thus, developing strategies to improve early diagnosis of adnexal malignancy is critical to improve the efficacy of treatment.

Gray-scale and Doppler ultrasonography is a convenient imaging modality for visualizing adnexal masses, with advantages such as cost-effectiveness and radiation-free safety (4, 5). However, there are many overlapping ultrasonic features between benign and malignant adnexal masses (6, 7). Furthermore, although color Doppler flow imaging can provide helpful information about blood flow in adnexal masses, it has limitations such as low sensitivity to display slow or deeply located blood flow vessels.

Tumor angiogenesis is necessary for tumor growth and is an independent prognostic indicator for survival in cancer patients, such as in ovarian carcinoma (8, 9). Advances in contrast enhanced ultrasonography (CEU) have enabled the characterization of tumor vascularity, such as in hepatic (10, 11), breast (12, 13), gastric (14), prostate (15), and cardiac masses (16). Several studies have reported that qualitative (17) and quantitative (18–20) CEU can improve the performance of sonography in distinguishing benign adnexal masses from malignancy. However, several factors impact CEU parameter values such as respiration, depth of mass, heart rate, and patient characteristics. Contrast intensity is also affected by contrast agent dosage, administration speed and instrument setting. Therefore, the primary objective of this study was to evaluate the usefulness of corrected CEU parameters in the differential diagnosis of adnexal masses.

## Methods

### Study population

Between January 2021 and December 2021, we prospectively studied 51 consecutive patients (40 ± 14 years old, range: 19–78 years old) with adnexal masses. All patients underwent a conventional ultrasonography examination and CEU. The inclusion criteria were as follows: (1) patients with ultrasound diagnosis of unilocular-solid (a single cyst without septa and without solid parts or papillary excrescences), multilocular-solid (a cyst with at least one septum but no solid parts or papillary excrescences) or a unilocular solid cyst (a single cyst containing solid parts or papillary excrescences but no septa), a multi-locular solid cyst (a cyst with at least one septum and solid parts or papillary excrescences), or a solid tumor (a tumor with solid components in 80% or more of the tumor), solid adnexal mass, or multi-locular adnexal cyst (21); and (2) patients with pathology results obtained from a surgical specimen within three months of surgery. Exclusion criteria were as follows: refusal to sign informed consent forms, severe renal insufficiency, right-to-left shunt heart disease syndrome, pregnancy or lactation, and age less than 18 years. Borderline tumors were classified as malignant.

This study was approved by the ethics committees of Guangzhou Women and Children Medical Center (approval number # 194A01) and the Second Affiliated Hospital of Zhejiang University School of Medicine (approval number # 0741). Written informed consent was obtained from all patients before CEU.

### Conventional ultrasonography and CEU imaging

Conventional ultrasonography and transabdominal CEU examinations were performed with a commercially available ultrasound machine (Mindray, China) and an M3S transducer SC5-1U with a transmission frequency of 1.2–6.0 MHz. All gray scale and color Doppler images were acquired by a radiologist with more than 5 years of experience through transabdominal and transvaginal ultrasonography examination. The location, size, shape, internal echogenicity of the mass, peritoneal effusion, and vascularity were recorded, and the patients then underwent CEU. The vascularity in the adnexal mass was assessed according to International Ovarian Tumor Analysis color Doppler scoring system as follows: 1=no vascularization, 2=minimal vascularization, 3=moderate vascularization, and 4=high vascularization (21). Uterus and adnexal masses were simultaneously imaged in the same plane using real-time CEU preset with coded pulse inversion technique after bolus intravenous infusion of 1.5 ml SonoVue (Brocco, Geneva, Switzerland) through the antecubital vein. To reduce microbubble destruction, we preset the mechanical index (MI) to a low MI setting of 0.082. Image depth was adjusted to 8–12 cm according to the location of the adnexal mass. Time gain compensation was adjusted to achieve a homogeneous signal intensity of the mass. All settings were kept constant throughout each examination.

The target lesion was observed continuously for 2–3 min after bolus injection of 1.5 ml contrast agents. The real-time contrast perfusion cine loop was digitally stored for subsequent analysis. Patients were observed for complications for 30 min before being permitted to leave.

### Imaging analysis

Contrast enhancement of adnexal masses after the injection of contrast agent was quantitatively analyzed by two independent radiologists who were blinded to the clinical information and the final diagnosis of the patients. A region of interest (ROI) was drawn both within the adnexal mass and adjacent to the myometrium. The time-intensity curve (TIC) and contrast parameters of ROI, including base intensity (BI), arrival time (AT), peak intensity (PI), time to peak intensity (TTP), ascending slope (AS), and descending slope (DS), were obtained automatically with quantitative imaging analysis software. To reduce the impact of contrast agent dosage and injection speed on CEU parameters, corrected (TTP−AT) _mass/_(TTP−AT) _uterus_ and (PI−BI) _mass_/(PI−BI) _uterus_ were calculated. All lesions were confirmed by pathologic examination of surgical specimens.

### Statistical analysis

Data analysis was performed with SPSS version 26 (SPSS Inc.) and Graph Pad Prism version 8.0 (Graph Pad, La Jolla, CA, USA). Continuous variables were expressed as mean ± standard deviation. Two groups were compared using a two-sample t-test (continuous variables with normal distribution) or a Wilcoxon-Mann-Whitney test (continuous variables with non-normal distribution or ordinal parameters). Categorical data comparisons were analyzed with Pearson chi-squared tests or Fisher’s exact test. Receiver operating characteristic (ROC) curves were generated for each CEU parameter. The optimal cut-off value was determined as the point at which the Youden index (sensitivity + specificity-1) was maximal. The intra- and interobserver reproducibility of the measurements were evaluated by the intraclass correlation coefficients (ICCs) using a two-way mixed-effects model and a two-way random-effects model. ICC>0.80 and ICC = 0.60–0.80 were considered excellent and good, respectively. *P*<0.05 was considered to indicate statistical significance.

## Results

### Patient characteristics

**Figure 1:**
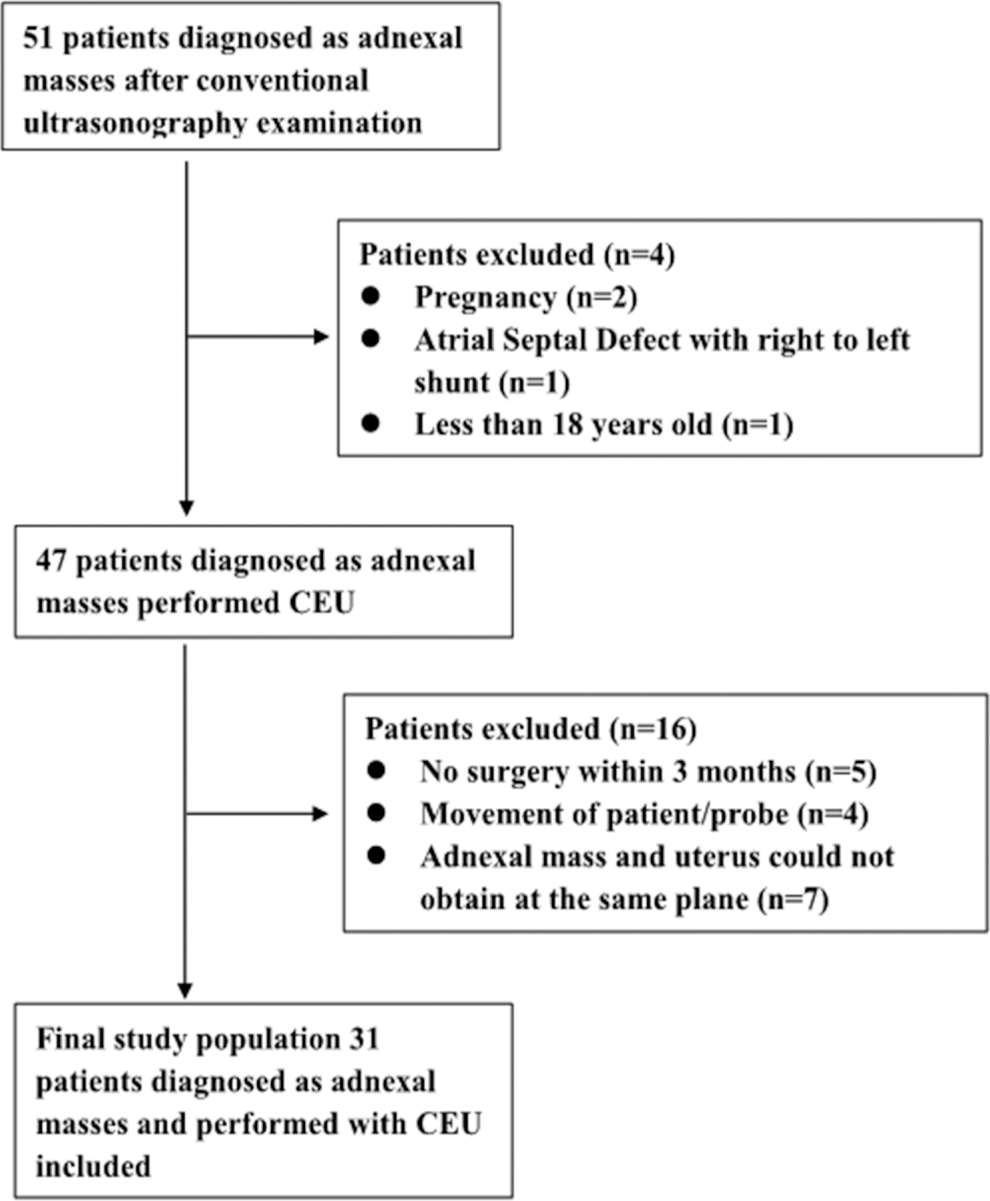
Flowchart for selection of patients with adnexal mass. In total, 31 out of 51 patients were included according to the selection criteria.

From January 2021 to November 2021, 51 patients with adnexal masses and conventional transabdominal or transvaginal ultrasonography examination in Guangzhou Women and Children Medical Center and the Second Affiliated Hospital Zhejiang University School of Medicine were initially enrolled. Among the 51 patients, 20 cases were excluded. The flowchart for patient selection for this study is illustrated in **Figure 1**. The final study group included 31 patients with 35 adnexal lesions including 20 (57.10%) benign lesions and 15 (42.90%) malignant lesions. All adnexal masses underwent histological verification, and characteristics are listed in **Table 1**. Eight patients with malignancy presented as asymptomatic; their adnexal masses were discovered incidentally during a routine medical examination.

**Table 1:** Pathological types of adnexal masses (n = 35).

Pathological type	n (%)
**Benign**	20 (57.14%)
Simple cyst	1 (2.86%)
Mesosalpinx cyst	1 (2.86%)
Mature teratoma	4 (11.43%)
Hydrosalpinx	1 (2.86%)
Serous cystadenoma	4 (11.43%)
Mucinous cystadenoma	2 (5.71%)
Fibrothecoma	1 (2.86%)
Endometrioma	5 (14.29%)
Brenner tumor	1 (2.86%)
**Malignant**	15 (42.86%)
Serous cystadenocarcinoma	3 (8.57%)
Mucinous cystadenocarcinoma	2 (5.71%)
Endometroid adenocarcinoma	2 (5.71%)
Granulosa cell tumor	1 (2.86%)
Spertoli-Leydig cell tumor	1 (2.86%)
Clear cell carcinoma	1 (2.86%)
Borderline cystadenoma	3 (8.57%)
Immature teratoma	2 (5.71%)

The clinical characteristics and laboratory results of the patients are summarized in **Table 2**. Peritoneal effusion and increased carbohydrate antigen 125 were more common in malignant masses compared with benign lesions, but the difference was not statistically significant (*P*=0.06 and 0.01, respectively).

**Table 2:** Clinical and conventional sonography characteristics of patients with adnexal masses (n = 35).

Characteristic	Benign (n = 20)	Malignant (n = 15)	*P* value
Age (year)	41.00 (32.50-51.50)	39.00 (30.50-63.00)	0.80
Postmenopausal (n)	5 (25.00%)	5 (33.30%)	0.71^a^
Maximum diameter of lesions (cm)	81.50 (68.00-101.50)	84.00 (37.00-114.50)	0.91
Bilateral (n)	3 (15.00%)	1 (6.70%)	0.62^a^
Peritoneal effusion (n)	3 (15.00%)	7 (46.70%)	0.06^a^
Color score			0.01
1	12 (60.00%)	3 (20.00%)	
2	6 (30.00%)	7 (46.67%)	
3	2 (10.00%)	3 (20.00%)	
4	0 (0%)	2 (13.33%)	
RI	0.56 (0.51-0.68)	0.45 (0.38-0.48)	0.01
CA-125 (u/ml)	26.45 (16.60-71.15)	74.70 (53.20-283.10)	0.01
HE-4 (pmol/L)	38.40 (29.35-52.75)	35.90 (35.35-81.35)	0.24

RI, resistance index; CA-125, carbohydrate antigen 125; HE-4, human epididymis protein-4.^a^Calculated with Fisher’s exact test.

### Conventional sonography findings

The conventional ultrasound features of the 35 adnexal masses including maximum diameter of lesions, color score, and resistance index (RI) value of vascularization in masses are listed in **Table 2**. There was no statistically significant difference in the maximum diameter of lesions between malignant and benign groups. The color score and RI of the vascularization of adnexal tumors were significantly different between the malignant and benign groups (both *P* = 0.01).

### Differential diagnostic ability of CEU with quantitative analysis

**Figure 2:**
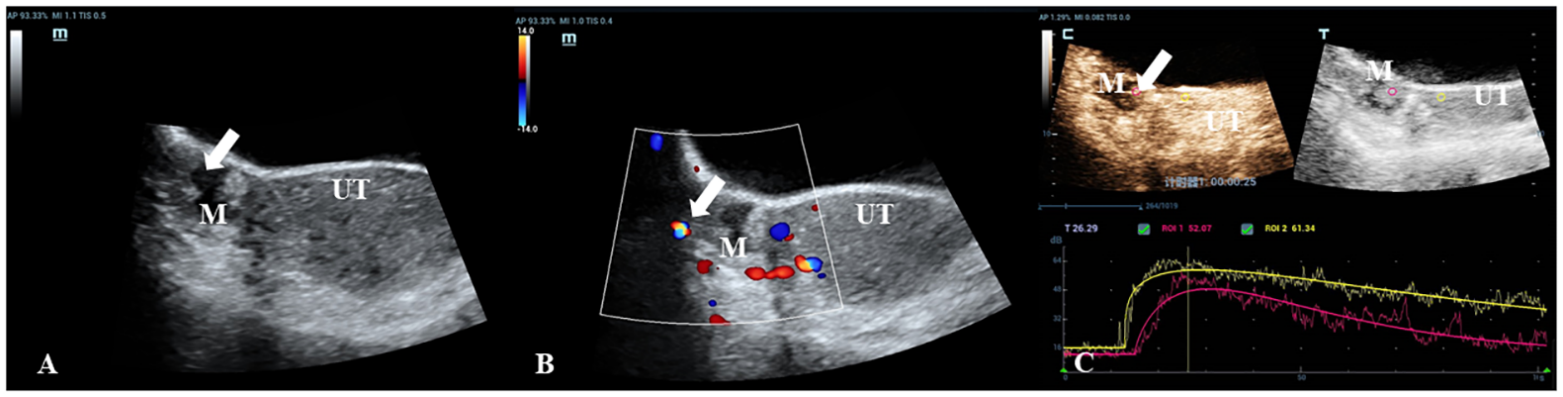
A 30-year-old woman with a pathologically proven borderline cystadenoma. **(A)** Transabdominal gray-scale ultrasound shows an 18-mm cystic mass (M) with a small 13 x 8mm papillary component at the right of uterus (UT). **(B)** Color Doppler reveals no blood flow signal in the papillary portion (color score=1). **(C)** CEU curve demonstrates that the tumor showed hypoenhancement in initial perfusion and faster washout compared with myometrium (mass: red, myometrium: yellow).

**Figure 3:**
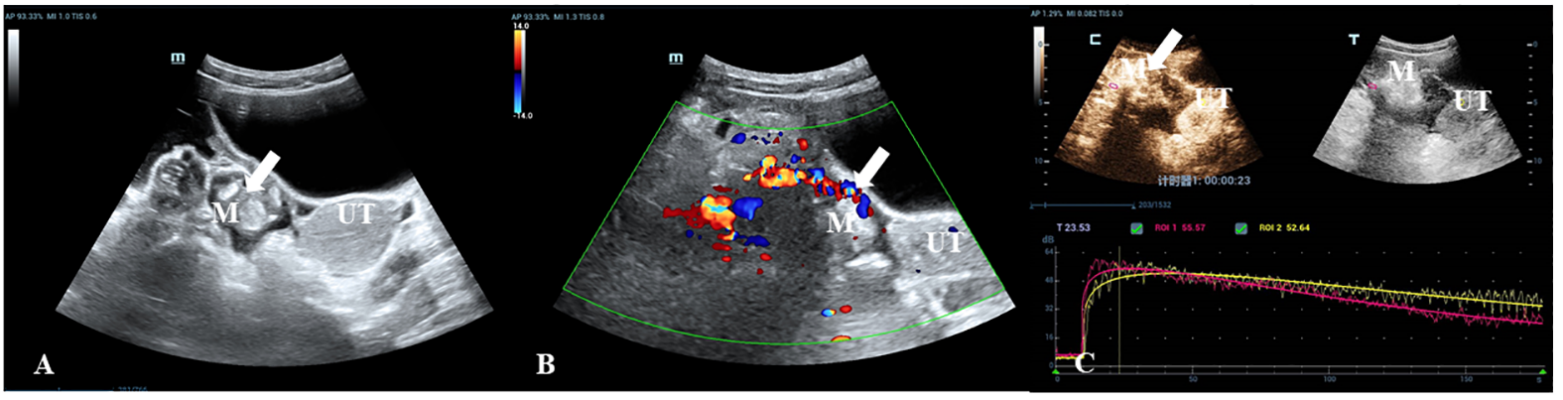
A 35-year-old woman with a pathologically proven immature teratoma. **(A)** Transabdominal gray-scale ultrasound shows a 30-mm complex cystic-solid mass (M) in the right adnexa. **(B)** Color Doppler reveals the color flow within the mass (color score=4). **(C)** CEU curve demonstrates that the tumor showed hyperenhancement in initial perfusion and faster washout compared with myometrium (mass: red, myometrium: yellow).

As shown in **Table 3**, the corrected CEU quantitative parameters in lesions of (TTP − AT) _mass/_(TTP − AT) _uterus_ and (PI − BI) _mass_/(PI − BI) _uterus_ were statistically different between malignant and benign adnexal mass groups (both *P*<0.05). The AS in the malignant tumor group was also significantly greater than that in benign tumors (**Figures 2**, **3**). The DS in the malignant group tended to be higher than that in benign masses but did not reach a statistically significant difference. Using ROC curve analysis, the optimal cut-off value for (TTP − AT) _mass/_(TTP − AT) _uterus_, AS, and (PI − BI) _mass_/(PI − BI) _uterus_, DS, TTP_mass_, and PI_mass_ for differentiating malignant adnexal masses from benign tumors were 1.05 (area under the curve (AUC): 0.93, *P*<0.05), 1.11 (AUC: 0.83, *P*<0.05), 0.82 (AUC: 0.73, *P*<0.05), −0.27(AUC: 0.66, *P*=0.16), 29.50(AUC:0.69, *P*=0.06), and 50.69(AUC:0.68, P=0.07) with a sensitivity and specificity of 93.33% and 85.00%, 86.67% and 75.00%, 86.67% and 60.00%, and 54.55% and 66.67%, 60% and 80%, 73.33% and 60.00% respectively (**Table 4**).

**Table 3:** Comparison of CEU parameters between benign and malignant masses.

Parameter	Benign tumor	Malignant tumor	*P* value
(TTP − AT)_mass/_(TTP − AT)_uterus_	1.48 (1.29–1.63)	0.72 (0.67–0.98)	<0.05
(PI − BI)_mass_/(PI − BI)_uterus_	0.74 (0.56–1.02)	1.04 (0.87–1.06)	<0.05
AS	0.96 (0.59–1.11)	1.29 (1.16–1.54)	<0.05
DS	-0.23 (-0.28– -0.16)	-0.28 (-0.36– -0.21)	0.16

Data are shown as mean (range), CEU, contrast enhanced ultrasonography; TTP, time to peak intensity; AT, arrival time; PI, peak intensity; BI, base intensity; AS, ascending slope; DS, descending slope.

**Table 4:** ROC curve analysis of the predicted probability of CEU parameters for evaluation of benign and malignant adnexal mass.

Parameter	Sensitivity	Specificity	Cut-off value	AUC	Std. error	*P* value	95% confidence interval	
							Lower limit	Upper limit
(TTP − AT)mass/(TTP − AT)uterus	93.33%	85.00%	1.05	0.93	0.04	<0.05	0.84	1
AS	86.67%	75.00%	1.11	0.83	0.07	<0.05	0.7	0.97
(PI − BI)mass/(PI − BI)uterus	86.67%	60.00%	0.82	0.73	0.09	<0.05	0.56	0.9
DS	54.55%	66.67%	-0.27	0.66	0.11	0.16	0.45	0.86
TTP mass	60.00%	80.00%	29.50	0.69	0.09	0.06	0.50	0.87
PI mass	73.33%	60.00%	50.69	0.68	0.09	0.07	0.49	0.86

ROC, receive operating characteristic; CEU, contrast enhanced ultrasonography; TTP, time to peak intensity; AT, arrival time; PI, peak intensity; BI, base intensity; AS, ascending slope; DS, descending slop.

### Inter-observer and intra-observer reproducibility

As shown in **Table 5**, the ICC for the same observer ranged from 0.93 (95% CI, 0.65–0.97) to 0.99 (95% CI, 0.98–0.99). The ICC between the two observers ranged from 0.88(95% CI, 0.60–0.95) to 0.99 (95% CI, 0.97–0.99).

**Table 5:** Intraobserver and interobserver reliability of CEU parameters.

Parameters	Intraobserver		Interobserver	
	ICC	95% CI	ICC	95% CI
BI	0.97	0.88–0.99	0.95	0.83–0.99
AT	0.93	0.75–0.98	0.88	0.60–0.97
TTP	0.99	0.97–0.99	0.99	0.95–0.99
PI	0.95	0.78–0.99	0.94	0.79–0.99
AS	0.91	0.65–0.98	0.89	0.61–0.97
DS	0.95	0.81–0.99	0.97	0.61–0.99

CEU, contrast enhanced ultrasonography; ICC, intraclass correlation; CI, confidence interval; BI, base intensity; AT, arrival time; TTP, time to peak intensity; PI, peak intensity; AS, ascending slope; DS, descending slope.

## Discussion

Our study results showed that the corrected quantitative visual temporal CEU parameters were statistically different between malignant adnexal masses and benign tumors. The values of (TTP − AT) _mass/_(TTP − AT) _uterus_, AS, and (PI − BI) _mass_/(PI − BI) _uterus_ had high diagnostic accuracy in distinguishing benign adnexal lesions from malignant tumors. The usefulness of CEU parameters demonstrated in this study provides practical differential diagnosis value of adnexal lesions with high reliability for radiologists.

Numerous efforts and international studies, such as International Ovarian Tumor Analysis simple rules (22), Gynecologic Imaging Reporting and Data System (23), and Ovarian-Adnexal Reporting and Data System (24), have been conducted to improve the ability of ultrasonography imaging for the diagnosis of adnexal masses. The diagnostic accuracy of malignant tumors has been enhanced by the combination of gray-scale ultrasound morphology and color Doppler flow imaging information (25). However, evaluation of gray-scale and Doppler ultrasound examination of adnexal masses is dependent on experience. Furthermore, malignant and benign lesions show overlapping features on gray-scale morphology and blood flow features (26).

Angiogenesis is a prerequisite for the growth of malignant tumors and an early event during tumor development (27). Microvessel density (MVD) influences the nutritional status of tumors and facilitates tumor growth, proliferation and invasion. MVD is associated with a poorer prognosis in breast and kidney cancer patients (27, 28) and correlates with the depth of tumor invasion (29). Intravenous CEU has been used widely to assess tumor angiogenesis *in vivo (* 10–20) and provides detailed information about the vascularity and blood flow kinetics in normal and pathologic tissues. Previous studies have shown that malignant ovarian masses generally have a greater PI compared with benign masses (17–20). However, Li et al. calculated PI − BI for quantitative analysis of the microvasculature. The authors revealed that PI − BI in carcinoma tissues was significantly higher than that in normal or benign tissues (*P*<0.001) and demonstrated that PI − BI corresponds with MVD, which was calculated by counting CD34-positive vascular endothelial cells (r=0.921, *P*<0.001) (29). Tang et al. showed that visual assessment of the degree of enhancement of cardiac masses to the adjacent myocardium during contrast perfusion echocardiography had high diagnostic accuracy for the differentiation of benign tumors from malignant tumors, with a sensitivity and specificity of 100% and 97%, respectively (16). In our study, we found that (PI − BI) _mass_/(PI − BI) _uterus_ was significantly higher in the adnexal carcinoma group than that in the benign group (*P*<0.05). ROC curve analysis revealed that the sensitivity and specificity of (PI − BI) _mass_/(PI − BI) _uterus_ in differentiating benign lesions from malignancy were 86.67% and 60.00%, respectively (cut-off: 0.82, AUC: 0.73, *P*<0.05). The specificity was only 60.00%, indicating substantial overlap between the benign and malignant tumors, especially between benign and borderline tumors.

Furthermore, the temporal features AT and TTP are affected by patient heart rate and cardiac function as well as by the velocity of bolus injection. To reduce these individual factors impacting CEU parameters, we measured (TTP − AT) _mass/_(TTP − AT) _uterus_. We found that (TTP − AT) _mass/_(TTP − AT) _uterus_ was significantly smaller in the malignant tumor group than in the benign tumor group. The result was consistent with the report by Sconfienza (30), in which the authors used the absolute value of TTP. We also found that (TTP − AT) _mass/_(TTP − AT) _uterus_ performed better with higher accuracy than the other parameters in distinguishing between benign adnexal masses and malignant cases. When the optimal cut-off was 1.05, the sensitivity, specificity, and AUC were 93.33%, 85.00%, and 0.93, respectively (*P*<0.05).

The other kinetics CEU parameter AS for malignant tumors was significantly greater than that for benign lesion. To our knowledge, no CEU study has used AS to distinguish malignant adnexal tumors from benign one. Our result was consistent with the findings reported by Kazerooni, who applied dynamic contrast-enhanced MRI to classify adnexal masses (31). Parameters like wash-out time could help differentiate benign and malignant tumors and even be more accurate than Doppler sonography for the discrimination of adnexal cancer from benign tumors. However, the kinetics CEU parameter DS that reflects wash-out of vascularity did not show a significant difference between malignant tumors and benign tumors in this study, indicating that the results obtained with this approach show a wide variability (18, 32). The tumor vasculature exhibits atypical morphological features and is characterized by dilated, tortuous disorganized blood vessels, arteriovenous fistula, and incomplete muscularization of vessel walls. This results in lower resistance to flow, few systolic-diastolic variations in blood flow velocity, and shorter wash-out time compared with that in normal vessels (27, 28, 33).

This study has several limitations. First, the number of cases included in this study is small, and we could not analyze the differences in the subtypes of adnexal mass, since there are some overlaps in different lesions. Second, we did not compare the difference between absolute and corrected CEU parameters in this study. However, other authors have previously studied the absolute CEU parameters.

## Conclusions

Malignant and benign adnexal tumors have different degrees of the kinetics of CEU parameters. We showed that both visual assessment and temporal assessment of the degree of enhancement of adnexal masses to adjacent myometrium after administration of contrast agents had high diagnostic accuracy in the discrimination of benign tumors from malignant tumors.

## Data availability statement

The datasets presented in this study can be found in online repositories. The names of the repository/repositories and accession number(s) can be found in the article/**Supplementary Material**.

## Ethics statement

The studies involving human participants were reviewed and approved by the ethics committees of Guangzhou Women and Children Medical Center and the Second Affiliated Hospital of Zhejiang University School of Medicine. Written informed consent was obtained from all patients before CEU. The patients/participants provided their written informed consent to participate in this study.Written informed consent was obtained from the individual(s) for the publication of any potentially identifiable images or data included in this article.

## Author contributions

Conception and design: WS, HW, and JG. Administrative support: HW. Provision of materials or patients: WS, QF, and CL. Collection and assembly of data: YZ, YW and WZ. Data analysis and interpretation: ZL, YW, FW and CML. Manuscript Writing: WS and YC. Final approval of manuscript: All the authors contributed

## Funding

We thank Funding of Guangzhou Institute of Pediatrics/Guangzhou Women and Chindren’s Medical Center (grant number: 4001022) for supporting the manuscript preparation and publication.

## Acknowledgments

We are grateful to Youbin Deng, MD (Department of Medical Ultrasonics, Tongji Hospital, Tongji Medical College, Huazhong University of Science & Technology, Wuhan, China), for writing assistance and review of the English in the manuscript.

## Conflict of interest

The authors declare that the research was conducted in the absence of any commercial or financial relationship that could be construed as a potential conflict of interest.

## Publisher’s note

All claims expressed in this article are solely those of the authors and do not necessarily represent those of their affiliated organizations, or those of the publisher, the editors and the reviewers. Any product that may be evaluated in this article, or claim that may be made by its manufacturer, is not guaranteed or endorsed by the publisher.
